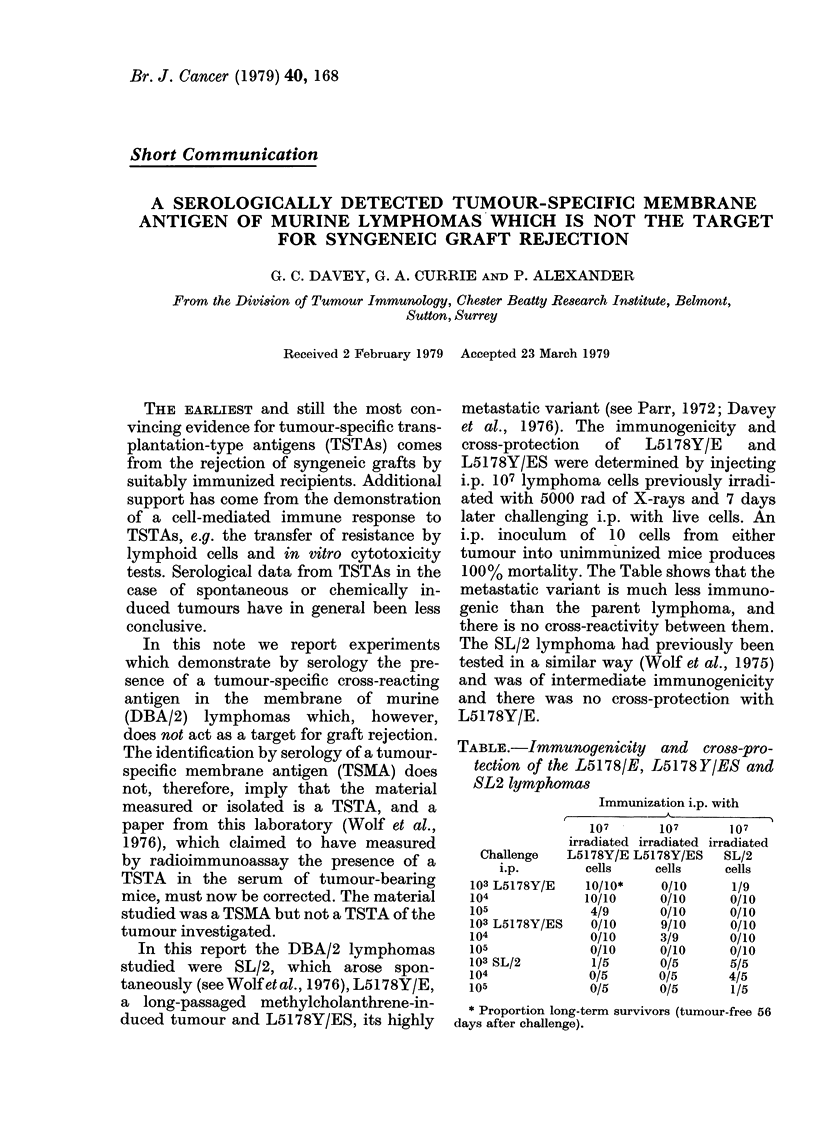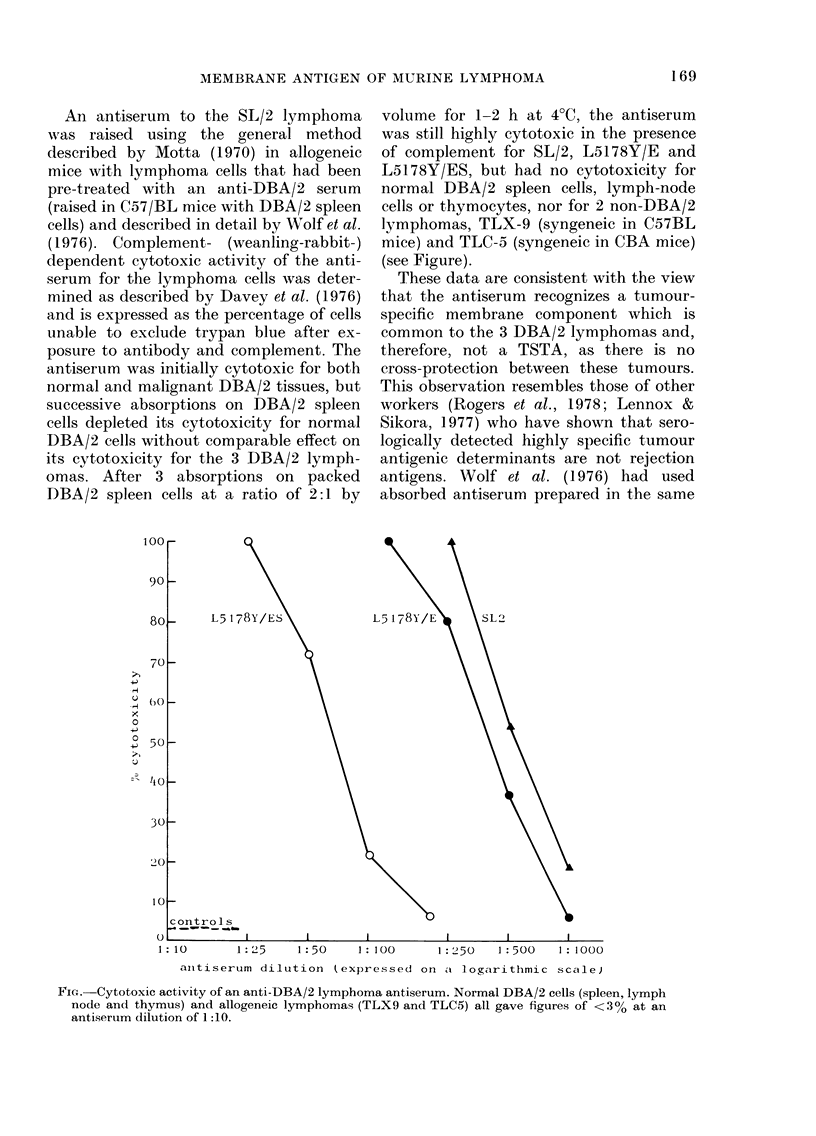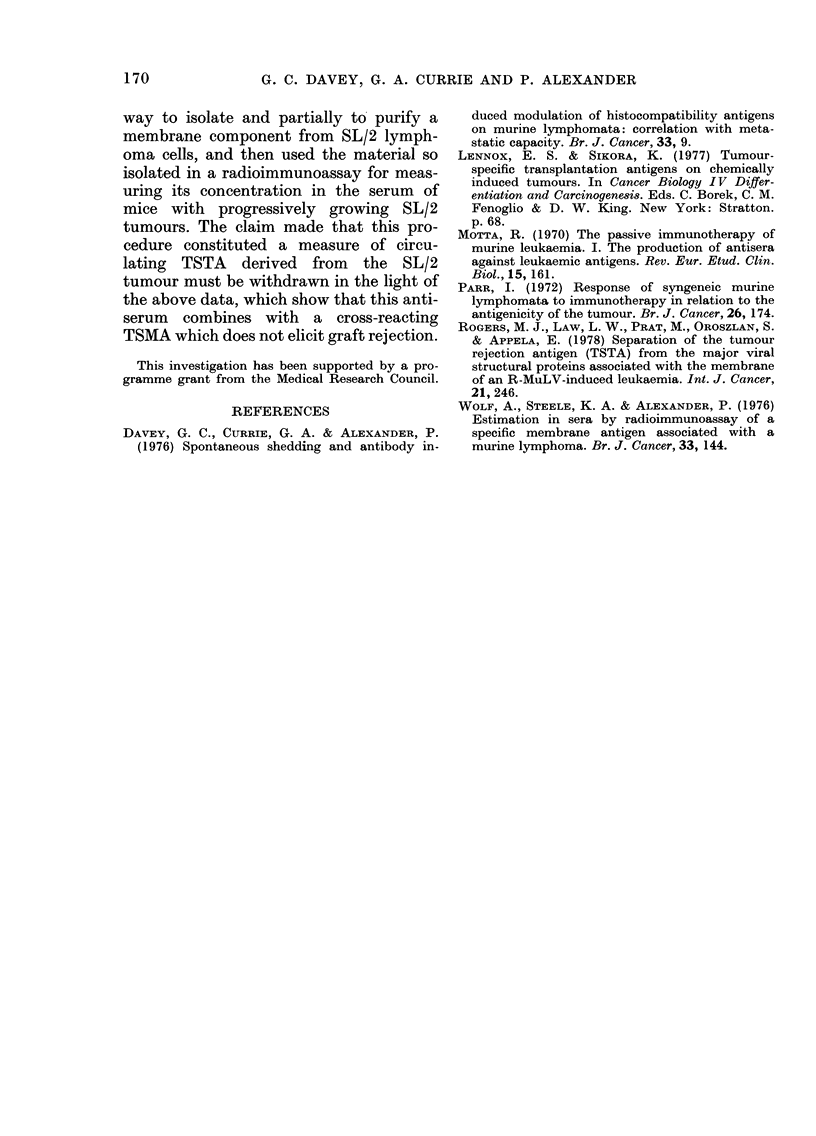# A serologically detected tumour-specific membrane antigen of murine lymphomas which is not the target for syngeneic graft rejection.

**DOI:** 10.1038/bjc.1979.155

**Published:** 1979-07

**Authors:** G. C. Davey, G. A. Currie, P. Alexander


*Br. J. Cancer* (1979) **40**, 168

### Short Communication

### A SEROLOGICALLY DETECTED TUMOUR-SPECIFIC MEMBRANE ANTIGEN OF MURINE LYMPHOMAS WHICH IS NOT THE TARGET FOR SYNGENEIC GRAFT REJECTION

G. C. DAVEY, G. A. CURRIE AND P. ALEXANDER

*From the Division of Tumour Immunology, Chester Beatty Research Institute, Belmont, Sutton, Surrey*

Received 2 February 1979 Accepted 23 March 1979

THE EARLIEST and still the most convincing evidence for tumour-specific transplantation-type antigens (TSTAs) comes from the rejection of syngeneic grafts by suitably immunized recipients. Additional support has come from the demonstration of a cell-mediated immune response to TSTAs, *e.g.* the transfer of resistance by lymphoid cells and *in vitro* cytotoxicity tests. Serological data from TSTAs in the case of spontaneous or chemically induced tumours have in general been less conclusive.

In this note we report experiments which demonstrate by serology the presence of a tumour-specific cross-reacting antigen in the membrane of murine (DBA/2) lymphomas which, however, does *not* act as a target for graft rejection. The identification by serology of a tumour-specific membrane antigen (TSMA) does not, therefore, imply that the material measured or isolated is a TSTA, and a paper from this laboratory (Wolf *et al.*, 1976), which claimed to have measured by radioimmunoassay the presence of a TSTA in the serum of tumour-bearing mice, must now be corrected. The material studied was a TSMA but not a TSTA of the tumour investigated.

In this report the DBA/2 lymphomas studied were SL/2, which arose spontaneously (see Wolf *et al.*, 1976), L5178Y/E, a long-passaged methylcholanthrene-induced tumour and L5178Y/ES, its highly metastatic variant (see Parr, 1972; Davey *et al.*, 1976). The immunogenicity and cross-protection of L5178Y/E and L5178Y/ES were determined by injecting i.p. $10^7$ lymphoma cells previously irradiated with 5000 rad of X-rays and 7 days later challenging i.p. with live cells. An i.p. inoculum of 10 cells from either tumour into unimmunized mice produces 100% mortality. The Table shows that the metastatic variant is much less immunogenic than the parent lymphoma, and there is no cross-reactivity between them. The SL/2 lymphoma had previously been tested in a similar way (Wolf *et al.*, 1975) and was of intermediate immunogenicity and there was no cross-protection with L5178Y/E.

TABLE.—*Immunogenicity and cross-protection of the L5178/E, L5178Y/ES and SL2 lymphomas*

| Challenge i.p. | Immunization i.p. with $10^7$ irradiated L5178Y/E cells | $10^7$ irradiated L5178Y/ES cells | $10^7$ irradiated SL/2 cells |
|---|---|---|---|
| $10^3$ L5178Y/E | 10/10* | 0/10 | 1/9 |
| $10^4$ | 10/10 | 0/10 | 0/10 |
| $10^5$ | 4/9 | 0/10 | 0/10 |
| $10^3$ L5178Y/ES | 0/10 | 9/10 | 0/10 |
| $10^4$ | 0/10 | 3/9 | 0/10 |
| $10^5$ | 0/10 | 0/10 | 0/10 |
| $10^3$ SL/2 | 1/5 | 0/5 | 5/5 |
| $10^4$ | 0/5 | 0/5 | 4/5 |
| $10^5$ | 0/5 | 0/5 | 1/5 |

* Proportion long-term survivors (tumour-free 56 days after challenge).

An antiserum to the SL/2 lymphoma was raised using the general method described by Motta (1970) in allogeneic mice with lymphoma cells that had been pre-treated with an anti-DBA/2 serum (raised in C57/BL mice with DBA/2 spleen cells) and described in detail by Wolf *et al.* (1976). Complement- (weanling-rabbit-) dependent cytotoxic activity of the antiserum for the lymphoma cells was determined as described by Davey *et al.* (1976) and is expressed as the percentage of cells unable to exclude trypan blue after exposure to antibody and complement. The antiserum was initially cytotoxic for both normal and malignant DBA/2 tissues, but successive absorptions on DBA/2 spleen cells depleted its cytotoxicity for normal DBA/2 cells without comparable effect on its cytotoxicity for the 3 DBA/2 lymphomas. After 3 absorptions on packed DBA/2 spleen cells at a ratio of 2:1 by volume for 1–2 h at 4°C, the antiserum was still highly cytotoxic in the presence of complement for SL/2, L5178Y/E and L5178Y/ES, but had no cytotoxicity for normal DBA/2 spleen cells, lymph-node cells or thymocytes, nor for 2 non-DBA/2 lymphomas, TLX-9 (syngeneic in C57BL mice) and TLC-5 (syngeneic in CBA mice) (see Figure).

These data are consistent with the view that the antiserum recognizes a tumour-specific membrane component which is common to the 3 DBA/2 lymphomas and, therefore, not a TSTA, as there is no cross-protection between these tumours. This observation resembles those of other workers (Rogers *et al.*, 1978; Lennox & Sikora, 1977) who have shown that serologically detected highly specific tumour antigenic determinants are not rejection antigens. Wolf *et al.* (1976) had used absorbed antiserum prepared in the same

FIG.—Cytotoxic activity of an anti-DBA/2 lymphoma antiserum. Normal DBA/2 cells (spleen, lymph node and thymus) and allogeneic lymphomas (TLX9 and TLC5) all gave figures of $<3\%$ at an antiserum dilution of 1:10.

way to isolate and partially to purify a membrane component from SL/2 lymphoma cells, and then used the material so isolated in a radioimmunoassay for measuring its concentration in the serum of mice with progressively growing SL/2 tumours. The claim made that this procedure constituted a measure of circulating TSTA derived from the SL/2 tumour must be withdrawn in the light of the above data, which show that this antiserum combines with a cross-reacting TSMA which does not elicit graft rejection.

This investigation has been supported by a programme grant from the Medical Research Council.

### REFERENCES

DAVEY, G. C., CURRIE, G. A. & ALEXANDER, P. (1976) Spontaneous shedding and antibody induced modulation of histocompatibility antigens on murine lymphomata: correlation with metastatic capacity. *Br. J. Cancer*, **33**, 9.

LENNOX, E. S. & SIKORA, K. (1977) Tumour-specific transplantation antigens on chemically induced tumours. In *Cancer Biology IV Differentiation and Carcinogenesis*. Eds. C. Borek, C. M. Fenoglio & D. W. King. New York: Stratton. p. 68.

MOTTA, R. (1970) The passive immunotherapy of murine leukaemia. I. The production of antisera against leukaemic antigens. *Rev. Eur. Etud. Clin. Biol.*, **15**, 161.

PARR, I. (1972) Response of syngeneic murine lymphomata to immunotherapy in relation to the antigenicity of the tumour. *Br. J. Cancer*, **26**, 174.

ROGERS, M. J., LAW, L. W., PRAT, M., OROSZLAN, S. & APPELA, E. (1978) Separation of the tumour rejection antigen (TSTA) from the major viral structural proteins associated with the membrane of an R-MuLV-induced leukaemia. *Int. J. Cancer*, **21**, 246.

WOLF, A., STEELE, K. A. & ALEXANDER, P. (1976) Estimation in sera by radioimmunoassay of a specific membrane antigen associated with a murine lymphoma. *Br. J. Cancer*, **33**, 144.